# Stress physiology of migrant birds during stopover in natural and anthropogenic woodland habitats of the Northern Prairie region

**DOI:** 10.1093/conphys/cou046

**Published:** 2014-10-11

**Authors:** Ming Liu, David L. Swanson

**Affiliations:** Department of Biology, University of South Dakota, Vermillion, SD 57069, USA

**Keywords:** Corticosterone, landbird migrants, migration, riparian habitat, stopover biology, stress response

## Abstract

Anthropogenic alterations of woodland habitat may influence stopover biology, which in turn could alter the stress physiology of migratory landbirds. Woodland stopover habitats are scarce in the Northern Prairie region of North America and consist of native riparian corridor woodlands (corridors) and smaller, more isolated woodlots of anthropogenic origin around farmsteads (woodlots). Corridor habitats have been greatly reduced since the time of European settlement, but woodlot habitats have appeared over this same time period. In this study, we compared stopover biology and stress physiology of migratory landbirds using natural and anthropogenic woodland habitats. We first tested for differences between birds in the two habitats for baseline corticosterone (CORT_B_) and the magnitude of the stress response for individual species, taxonomic families and foraging guilds. Plasma corticosterone increased significantly for all bird groups in both habitats following 30 min of restraint stress (CORT_30_), and neither CORT_B_ nor the magnitude of the stress response (CORT_30_ − CORT_B_) differed significantly between birds in the two habitats. Secondly, because CORT_B_ levels are often elevated and CORT secretion following a stressor is often suppressed for birds in poor body condition, we hypothesized that woodland migrants with higher fattening rates would show reduced CORT_B_ and a robust stress response. We tested this hypothesis by assessing the relationships between plasma corticosterone and plasma metabolites associated with refuelling. We found that CORT_B_ was negatively associated and the magnitude of the stress response positively associated with plasma triglycerides (an indicator of fat deposition), with opposite patterns for corticosterone and plasma β-hydroxybutyrate (an indicator of fat catabolism). These data suggest that both corridor and woodlot habitats serve as effective stopover habitat and that the reduction of corridor habitat and increased reliance on anthropogenic woodlots is not detrimental to the stress physiology of migrants in a region with limited woodland habitats.

## Abstract (precis)

This study addresses the question of whether increased anthropogenic influence on migration stopover sites/migration corridors affects the stress physiology of migratory landbirds. Our data suggest that the reduction of natural riparian corridor habitats and subsequent increased reliance of woodland migrants on anthropogenic woodlots for stopover is not detrimental to the stress physiology of these birds in a region with limited woodland habitats.

## Introduction

Migration is energetically expensive, and woodland migrant birds must refuel at stopover habitats along the migratory route (Moore and Woodrey, 1993; Yong and Moore, 1997; Moore and Aborn, 2000; Wikelski *et al.*, 2003; Faaborg *et al.*, 2010). Woodland habitats are scarce in the Northern Prairie region of North America and consist of natural riparian corridor woodlands (hereafter corridors) and small anthropogenic woodlots (hereafter woodlots; Castonguay, 1982; Swanson *et al.*, 2005; Gentry *et al.*, 2006). These natural corridors have been greatly reduced and degraded since the time of European settlement (Johnson *et al.*, 1976; Hesse, 1996; Dixon *et al.*, 2012). The reduction of corridor habitats, however, has occurred simultaneously with an increase in the number of small woodlots of anthropogenic origin (Bakker, 2003; Swanson *et al.*, 2005). Given that woodland migrants occupy both corridor and woodlot habitats in this region during migratory stopover (Martin, 1980; Dean, 1999; Bakker, 2003; Swanson *et al.*, 2003, 2005), these woodlots have the potential to provide a partial substitute for lost and degraded riparian woodlands as stopover habitat for woodland migrant birds.

One aspect of the biology of migratory birds that may be influenced by anthropogenic alterations to the natural landscape is their stress physiology, which can impact the timely and successful completion of migration, with potential fitness consequences (Heglund and Skagen, 2005; Carlisle *et al.*, 2009; Eikenaar *et al.*, 2014). Corticosterone (CORT) is the major glucocorticoid secreted in birds (Holmes and Phillips, 1976) and is a primary component of the stress response (Wingfield *et al.*, 1997). In general, plasma CORT levels are low prior to a stressor and increase rapidly (within minutes) in response to stress, reaching a peak after 30–60 min (e.g. Holberton *et al.*, 1996). The increase in plasma CORT in response to a stressor is known as the stress response and acts to direct the organism away from energetically costly activities, such as migration, breeding or courtship, to focus on activities that will promote immediate condition improvement and enhance the probability of survival, such as foraging (Wingfield *et al.*, 1992, 1995). Indeed, high plasma CORT may enhance fattening during migration (Astheimer *et al.*, 1992; Long and Holberton, 2004; Landys *et al.*, 2006; Lõhmus *et al.*, 2006; Holberton *et al.*, 2007, 2008; but see Eikenaar *et al.*, 2013, 2014). However, plasma CORT levels and stopover duration may show a U-shaped relationship, with migrants potentially having higher baseline CORT (CORT_B_) upon arrival at stopover sites, showing a decline as their body condition improves and an increase shortly before their departure, when fat stores are high (Piersma *et al.*, 2000; Landys-Ciannelli *et al.*, 2002; Eikenaar *et al.*, 2013). Such a U-shaped relationship between stopover duration and plasma CORT may still be consistent with CORT promoting fattening if fat migrants decrease foraging and fattening rate prior to departure (e.g. Loria and Moore, 1990), concurrent with elevated plasma CORT concentrations to stimulate departure. The elevated plasma CORT concentrations upon departure may function in relationship to other hormones as the cue to induce migratory flight (Schwabl *et al.*, 1991; Landys-Ciannelli *et al.*, 2002; Eikenaar *et al.*, 2013).

Plasma CORT levels in birds may be related to body condition, and many studies suggest that fat birds may show lower levels of CORT_B_ and a greater stress response than lean birds (Schwabl *et al.*, 1991; Marra and Holberton, 1998; Holberton, 1999; Jenni *et al.*, 2000; Piersma *et al.*, 2000; Mizrahi *et al.*, 2001; Landys-Ciannelli *et al.*, 2002; Long and Holberton, 2004; Raja-aho *et al.*, 2010; Fokidis *et al.*, 2011). Migrants in corridor and woodlot sites in the present study showed similar refuelling rates, as indicated by plasma metabolite analyses (Liu and Swanson, 2014), but whether the foraging effort required to achieve similar refuelling rates differs between corridors and woodlots is unknown. Highly fragmented landscapes, such as the woodlots in the present study, often reduce food availability and require increased foraging effort in comparison to more contiguous habitats, such as the corridor habitats in the present study (Ellis *et al.*, 2012). If differences in foraging effort exist between corridor and woodlot habitats, such differences could influence the stress physiology of migrants (Kitaysky *et al.*, 1999; Pravosudov *et al.*, 2001; Mark and Rubenstein, 2013), despite similar refuelling rates.

Differences in the size and structure of corridor and woodlot habitats could also affect other ecological factors, which could, in turn, influence stress physiology of migrant birds. In southeastern South Dakota, the average size of corridor woodlands is more than three times larger than woodlots, which results in much greater amounts of woodland edge for woodlot habitats, and corridors also harbour more diverse vegetation than woodlots (Castonguay, 1982; Gentry *et al.*, 2006; Liu and Swanson, 2014). These differences in size, structure and vegetative diversity of corridors and woodlots may produce differences in predation pressure, competition among migrants and thermal microclimates (Ellis *et al.*, 2012), all of which could potentially influence stress physiology. Predation pressure can alter the stopover biology of migrants (Lank *et al.*, 2003; Pomeroy, 2006; Taylor *et al.*, 2007) and influence the stress response (Cockrem and Silverin, 2002; Clinchy *et al.*, 2004; Newman *et al.*, 2013). Smaller woodland parcel size and increased edge facilitate the access of predators to smaller woodlands (Donovan *et al.*, 1997; Tewksbury *et al.*, 2006), so predation pressure is expected to be higher in woodlots than in corridors in our study area. Such differences in predation pressure could result in differences in stress physiology between migrants in corridors and woodlots in the present study.

Competition may also influence stress physiology, and better competitors, such as birds with better body condition or of larger size, may show lower CORT_B_ and a greater magnitude of the stress response (Lehnen and Krementz, 2005; Braasch *et al.*, 2014). Hatch-year birds are usually socially subordinate to adults, which may restrict the former's access to limited resources due to competitive interactions with adults (Moore *et al.*, 2003; Faaborg *et al.*, 2010). In addition, because juveniles in autumn are experiencing their first migration, foraging efficiency during stopover may be lower than that of experienced adults (Yong *et al.*, 1998; Heise and Moore, 2003). Consequently, the stress response might differ between ages, with higher CORT_B_ and a lower stress response in juveniles than in adults. The age ratios of migrants at woodlot and corridor study sites differ, with higher proportions of juveniles at woodlot sites, perhaps resulting from competition with adults (Dean *et al.*, 2004; Liu and Swanson, 2014); therefore, differences in stress physiology between birds in corridors and woodlots as a function of differing competitive interactions or differing age ratios might be expected.

Finally, the smaller size and increased edge of woodlots relative to corridors could affect thermal microclimates of birds in the two habitats. These changes would be likely to contribute to increased penetration of wind into woodlot habitats, which would function to reduce operative temperatures for birds in woodlots relative to corridors (Olson and Grubb, 2007). Such reduced temperatures may have metabolic consequences for woodland birds (Olson *et al.*, 2010), and thermal stress can influence the stress response in birds, particularly during periods of inclement weather (Wingfield *et al.*, 1983; Rogers *et al.*, 1993; Romero *et al.*, 2000). Thus, differences in microclimate could also contribute to differences in stress physiology for migrants in corridor and woodlot study sites.

In the present study, therefore, we tested for differences between birds in corridors and woodlots for CORT_B_ and the magnitude of the stress response for individual species, taxonomic families and foraging guilds to determine whether migrants in anthropogenic habitats demonstrate different stress physiology from those in natural riparian corridor habitats. As an additional objective, we also examined whether stress physiology in migrants was correlated with refuelling rates in the two habitats, as measured by plasma metabolites (Liu and Swanson, 2014).

## Materials and methods

### Study area

**Figure 1:**
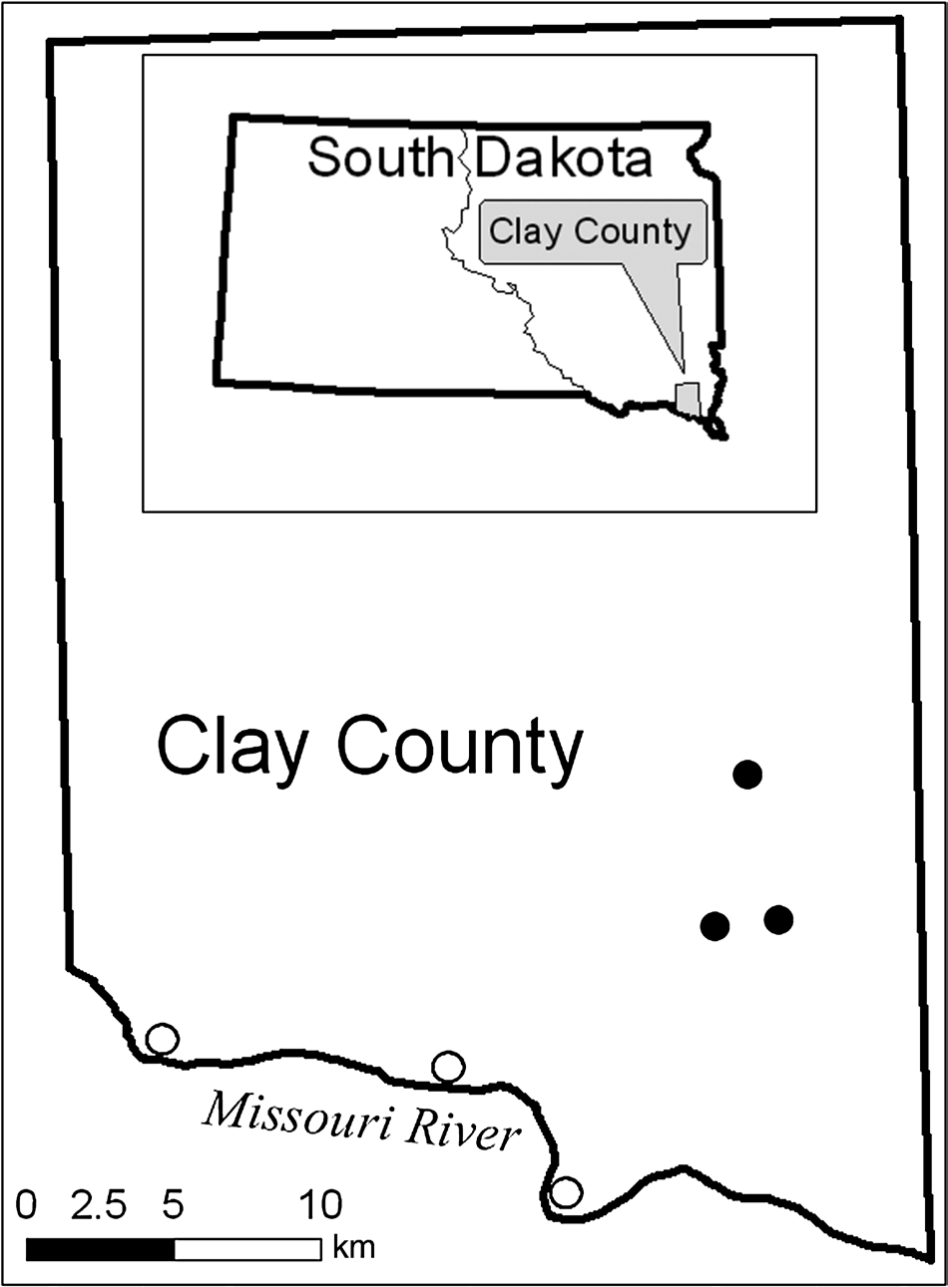
Study sites in Clay County, SD, USA. Open circles denote corridor sites and filled circles woodlot sites. Contiguous woodland areas for the study sites ranged from 1 to 1.6 km^2^ for corridor sites and from 0.17 to 0.2 km^2^ for woodlot sites.

This study was conducted in southeastern South Dakota, USA, where woodlands cover ∼4% of total land area and the Missouri River corridor provides the most extensive riparian woodlands in the region (Castonguay, 1982). Woodlots cover ∼1% of the total landscape area in southeastern South Dakota and they are smaller, more isolated and less vegetatively diverse than corridor woodlands (Castonguay, 1982; Swanson *et al.*, 2003, 2005). Corridor woodlands are mostly dominated by cottonwoods (*Populus deltoides*), with some later successional tree species, such as green ash (*Fraxinus pennsylvanica*), elm (*Ulmus* spp.), and hackberry (*Celtis occidentalis*), among other trees (Hesse, 1996; Swanson *et al.*, 2005; Dixon *et al.*, 2012). The woodlot study sites are comprised primarily of elm, mulberry (*Morus alba*), box elder (*Acer negundo*), hackberry and green ash (Swanson *et al.*, 2003; Gentry *et al.*, 2006). Three representative study sites were sampled from both corridor (all along the Missouri River) and woodlot habitat types in Clay County, South Dakota (Fig. 1), from sites sampled previously by Swanson *et al.* (2005) and Gentry *et al.* (2006). Woodlot and corridor study sites were separated by at least 9.5 km.

### Bird capture and blood sampling

Migrants were captured with 9 m, 36-mm-mesh, nylon mist nets. Each of the six study sites was sampled once per week, and we rotated through the sites in the same order, with woodlot and corridor sites sampled on alternating days during spring (mid-April to early June, 2010–2012) and autumn (mid-August to mid-October, 2010–2011) migration periods. Mist nets were operated for a few hours, beginning at sunrise, and during similar weather conditions, i.e. no rain and relatively low wind speed (<30 kph). We erected up to three nets per individual field worker at each site, and monitored nets continuously so that we could extract birds from the nets immediately upon capture. The initial blood samples for CORT_B_ determination were collected within 3 min of birds hitting the net. We collected blood samples with 50 µl heparinized capillary tubes via venipuncture from the brachial vein on the underside of the wing (Schwabl *et al.*, 1991; Wingfield *et al.*, 1992; Palm *et al.*, 2013). A second sample was taken 30 min after capture (CORT_30_), with birds held in cloth bags during the 30 min restraint stress (Wingfield *et al.*, 1992). Depending on bird body mass, a maximum of 100–200 µl blood was sampled from each individual bird (Voss *et al.*, 2010). After collection, blood samples were transferred into microcentrifuge tubes and stored on ice while in the field. Upon return to the laboratory, blood samples were centrifuged for 10 min at 4°C and 3000***g***. The plasma was then removed to new microtubes and immediately stored at −60°C until later measurement of plasma CORT.

### Bird banding and morphological measurements

Prior to release following blood sampling, we banded birds with United States Geological Survey aluminum leg bands, identified each bird to species, and aged birds (autumn only; using skull ossification, Pyle, 1997) as after hatch year (AHY) or hatch year (HY). The date and time of capture were recorded for each bird. Unflattened wing chord and tarsus length were measured with a wing ruler to the nearest 0.5 mm or with callipers to the nearest 0.1 mm. We measured body mass to the nearest 0.1 g with an electronic scale (Ohaus Model LS200) and scored visible fat in the furculum and abdomen on a 0–5 scale (Helms and Drury, 1960). Following blood sampling and morphometric measurements, birds were released at the site of capture. Bird capture, blood sampling and banding were carried out under a Master Banding Permit from the United States Geological Survey Bird Banding Laboratory to D.L.S. All methods in the study were approved by the Institutional Animal Care and Use Committee at the University of South Dakota (protocol # 84-03-11-14B).

### Plasma metabolite measurement

Plasma metabolite data are from Liu and Swanson (2014), who measured plasma concentrations of triglycerides (TRIG), glycerol and β-hydroxybutyrate (BUTY) from the same individual birds for which CORT was measured in the present study. We measured plasma metabolites with commercially available spectrophotometric assay kits (Sigma-Aldrich Corp., St Louis, MO, USA) using a Beckman DU-7400 spectrophotometer (Liu and Swanson, 2014). We did not obtain sufficient volumes of blood from all individuals for all plasma metabolite and CORT measurements, so we determined the relationships between plasma CORT and plasma metabolites from a subset of individuals, for which we had measurements of both CORT and plasma metabolites.

### Plasma CORT measurement

We measured plasma CORT concentrations with commercially available microplate spectrophotometric end-point assay kits (Enzo Life Sciences ADI-901-097; Assay Designs Inc., Ann Arbor, MI, USA), according to the manufacturer's instructions (Forster *et al.*, 2008). We distributed samples across assay plates evenly with respect to season and species to minimize effects of inter-assay variation on subsequent analyses. All samples, controls and corticosterone standards (0–2000 ng/ml) were run in duplicate. Plasma corticosterone levels were detected by absorbance of samples at 405 nm using an automated plate reader and KineticCalc Jr software (Bio-Tek Instruments, Wonooski, VT, USA). Absorbance values from samples were applied to the standard curve generated (*r*^2^ = 0.99), and CORT levels were expressed as nanograms per millilitre. Absorbance values were also used to calculate the percentage of maximal binding, which ranged from 17.94 to 23.56% (*n* = 21), and the percentage of non-specific binding, which ranged from 2.11 to 2.98% (*n* = 21). The minimal detection limit was 27.0 pg/ml. Inter-assay samples (pooled from blood plasma of multiple species) were included in each of the plasma CORT assays to calibrate measured CORT levels and to monitor variation among assays. Inter-assay variance (average variation among assays) was 6.28% and intra-assay variance (average variation between duplicates) 9.15%.

### Statistics

We performed all statistical analyses with SAS software (Version 9.3, SAS Institute Inc., Cary, NC, USA). Values of CORT_B_, CORT_30_ and the magnitude of the stress response (CORT_30_ − CORT_B_) were compared for individual species, taxonomic groups (species grouped by families) and foraging guilds (Martin and Finch, 1995; Liu and Swanson, 2014; Table 1), with sample sizes ≥10 in each habitat. *Post hoc* power analyses, assuming a power of 0.80 and an α value of 0.05, revealed that these sample sizes are sufficient to detect significant differences at moderate to large effect sizes (>0.40) for all statistical comparisons (Cohen, 1988). Data were log_10_ transformed prior to analyses to meet the assumptions of normality and equal variance. We present CORT data as mean values ± SEM (in nanograms per millilitre). We conducted the taxa and foraging guild comparisons to increase overall sample sizes for between-habitat comparisons and to include data from species for which we did not have sufficient sample sizes for individual species comparisons. We reasoned that, in general, species within the same taxonomic family or the same foraging guild would respond in a similar manner to potential variation in resources or other ecological factors (e.g. predation, microclimates) between corridor and woodlot habitats, so we pooled data from species in these groups, after controlling for species effects (see below), for between-habitat comparisons. Moreover, the conservation of woodland habitat for woodland migrants may need to focus on bird groups requiring similar resources or responding in a similar manner to other ecological factors (e.g. families or foraging guilds), in addition to individual species, so comparisons among taxa and foraging guilds may better inform conservation planning.

**Table 1:** Classification of foraging guild and taxa of migratory landbird species at our study sites

Guild	Taxon	Spring	Autumn
FGG		Yellow-rumped warbler (*Setophaga coronata*)	Mourning warbler (*Geothlypis philadelphia*)
		Common yellowthroat (*Geothlypis trichas*)	Yellow-rumped warbler (*S. coronata*)
	VIR	Yellow-throated vireo (*Vireo flavifrons*)	Yellow-throated vireo (*V. flavifrons*)
		Red-eyed vireo (*Vireo olivaceus*)	Blue-headed vireo (*Vireo solitarius*)
		Warbling vireo (*Vireo gilvus*)	Red-eyed vireo (*V. olivaceus*)
			Warbling vireo (*V. gilvus*)
GFG		Gray catbird (*Dumetella carolinensis*)	Gray catbird (*D. carolinensis*)
		Ovenbird (*Seiurus aurocapilla*)	Ovenbird (*S. aurocapilla*)
	THR	Northern waterthrush (*Parkesia noveboracensis*)	Northern waterthrush (*P. noveboracensis*)
		Wood thrush (*Hylocichla mustelina*)	Wood thrush (*H. mustelina*)
		Gray-checked thrush (*Catharus fuscescens*)	Swainson's thrush (*Catharus ustulatus*)
	SPA	Swainson's thrush (*C. ustulatus*)	Hermit thrush (*Catharus guttatus*)
		Lincoln's sparrow (*Melospiza lincolnii*)	Lincoln's sparrow (*M. lincolnii*)
		White-throated sparrow (*Zonotrichia albicollis*)	Harris's sparrow (*Zonotrichia querula*)
			White-throated sparrow (*Z. albicollis*)
			Dark-eyed junco (*Junco hyemalis*)
			Fox sparrow (*Passerella iliaca*)
			Indigo bunting (*Passerina cyanea*)
	FLY	Least flycatcher (*Empidonax minimus*)	
		Traill's flycatcher (*Empidonax alnorum* and *Empidonax traillii*)	
		Yellow-bellied flycatcher (*Empidonax flaviventris*)	

Abbreviations: FGG, foliage-gleaning guild; FLY, flycatchers; GFG, ground-foraging guild; SPA, sparrows; THR, thrushes; VIR, vireos.

To adjust for individual differences in body size, we used principal component analysis to incorporate structural measurements (wing chord and tarsus length) to produce a principal component (PC1) for each individual species describing body size (Pedhazur, 1997; Guglielmo *et al.*, 2005). We used a backward stepwise multiple regression approach to generate predictors with CORT_B_, CORT_30_ or the magnitude of the stress response as the dependent variable. Independent variables in the model included the principal component for body size, fat score (the average of furcular and abdominal fat scores), age (autumn only), season (foraging guilds only), capture time, capture date and year, with variables retained in the model at *P* < 0.10. The variables retained after multiple regressions were maintained as covariates in an analysis of covariance (ANCOVA) to test for differences between birds from corridors and woodlots. We applied ANCOVA (for CORT_B_ and CORT_30_) or repeated-measures ANCOVA (for the magnitude of the stress response) to test for differences between habitat types and ages (autumn only) in both spring and autumn. If no covariates were generated by multiple regressions, we used analysis of variance (ANOVA; for CORT_B_ and CORT_30_) or repeated-measures ANOVA (for the magnitude of the stress response) to test for differences between birds from corridors and woodlots. Repeated-measures ANOVA or ANCOVA also allowed us to test whether CORT_B_ and CORT_30_ differed significantly for all species, taxa and foraging guilds.

To control for possible individual species effects on plasma CORT levels in analyses of foraging guilds and taxa, we included species and habitat type as categorical independent variables, along with the species × habitat type interaction term, in the general linear model ANCOVA. The rationale behind this correction for species effects is that different species may show varying raw plasma CORT concentrations, so significant biases could exist in pooled species comparisons if species are combined without controlling for species differences. For individual species with sample sizes ≥10 in both corridors and woodlots, we also applied unpaired Student's *t*-tests to compare fat scores.

Finally, for correlation analyses between CORT (CORT_B_, magnitude of the stress response) and plasma metabolites (TRIG and BUTY) and between CORT_B_ and the magnitude of the stress response, we normalized data for individual birds to species averages (for species with *n* ≥ 10), so that we could pool data from individual birds in our comparisons. To normalize the CORT and plasma metabolite data for each individual bird, the individual values for CORT_B_, the magnitude of the stress response and plasma TRIG and BUTY levels were divided by the mean value for that species. These normalized data from individual birds were then used for correlation analyses of CORT_B_ and the magnitude of the stress response against TRIG and BUTY concentrations and of the magnitude of the stress response against CORT_B_.

## Results

### Comparisons of fat scores

No species showed significant between-habitat differences in fat scores, although the difference in fat scores for yellow-rumped warblers (*Setophaga coronata*) in autumn approached significance (*F*_1,51_ = 6.37, *P* = 0.08), with greater fat scores from corridors than from woodlots. We obtained sufficient sample sizes for comparisons between juveniles and adults only for warbling vireos (*Vireo gilvus*) and dark-eyed juncos (*Junco hyemalis*) in autumn, and fat scores were not statistically different between ages for these species.

### Between-habitat comparisons of CORT levels

**Figure 2:**
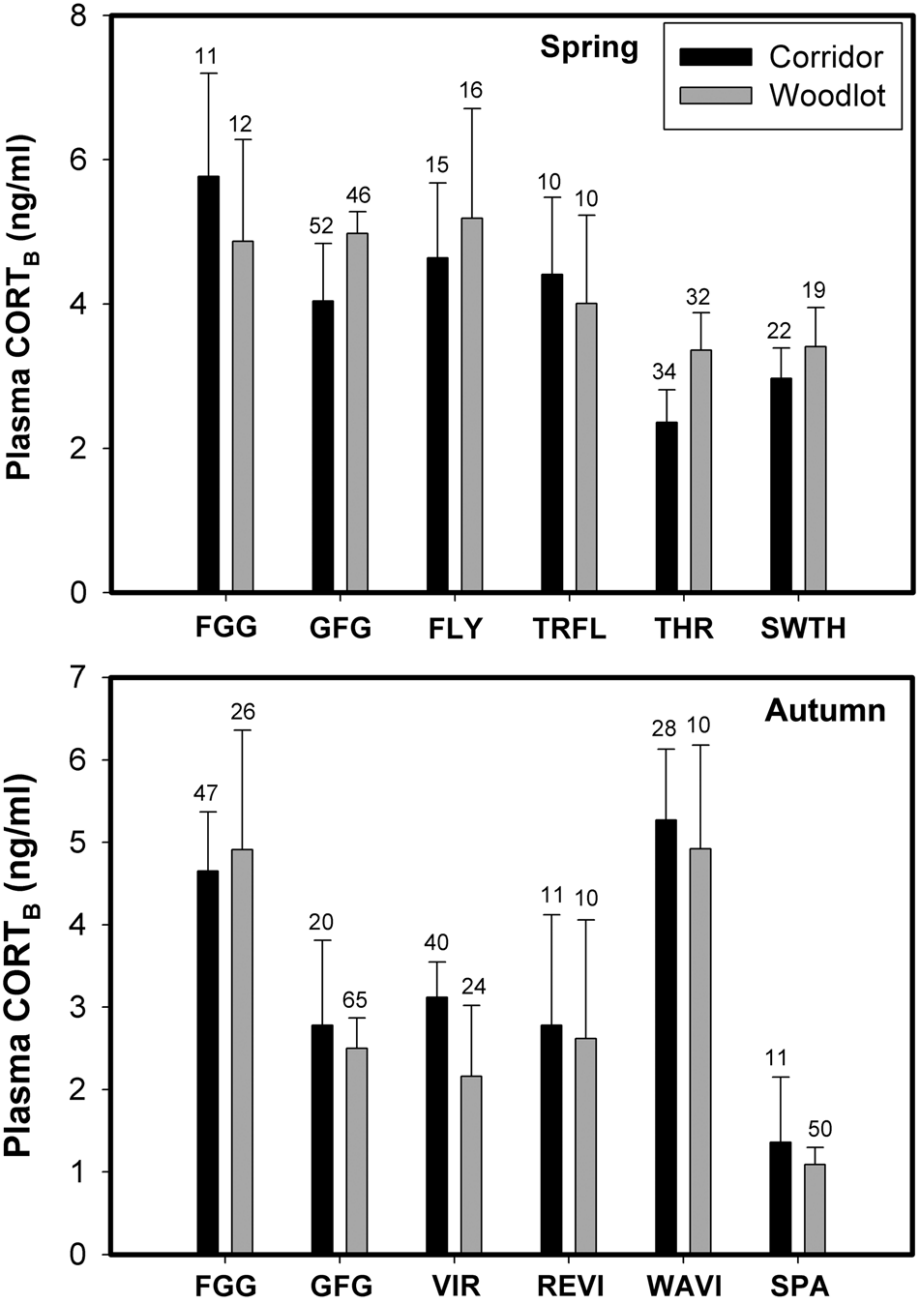
Mean (±SEM) baseline corticosterone (CORT_B_, in nanograms per millilitre) for spring and autumn individual species, taxa and foraging guilds. Abbreviations are as in Tables 1 and 2.

**Figure 3:**
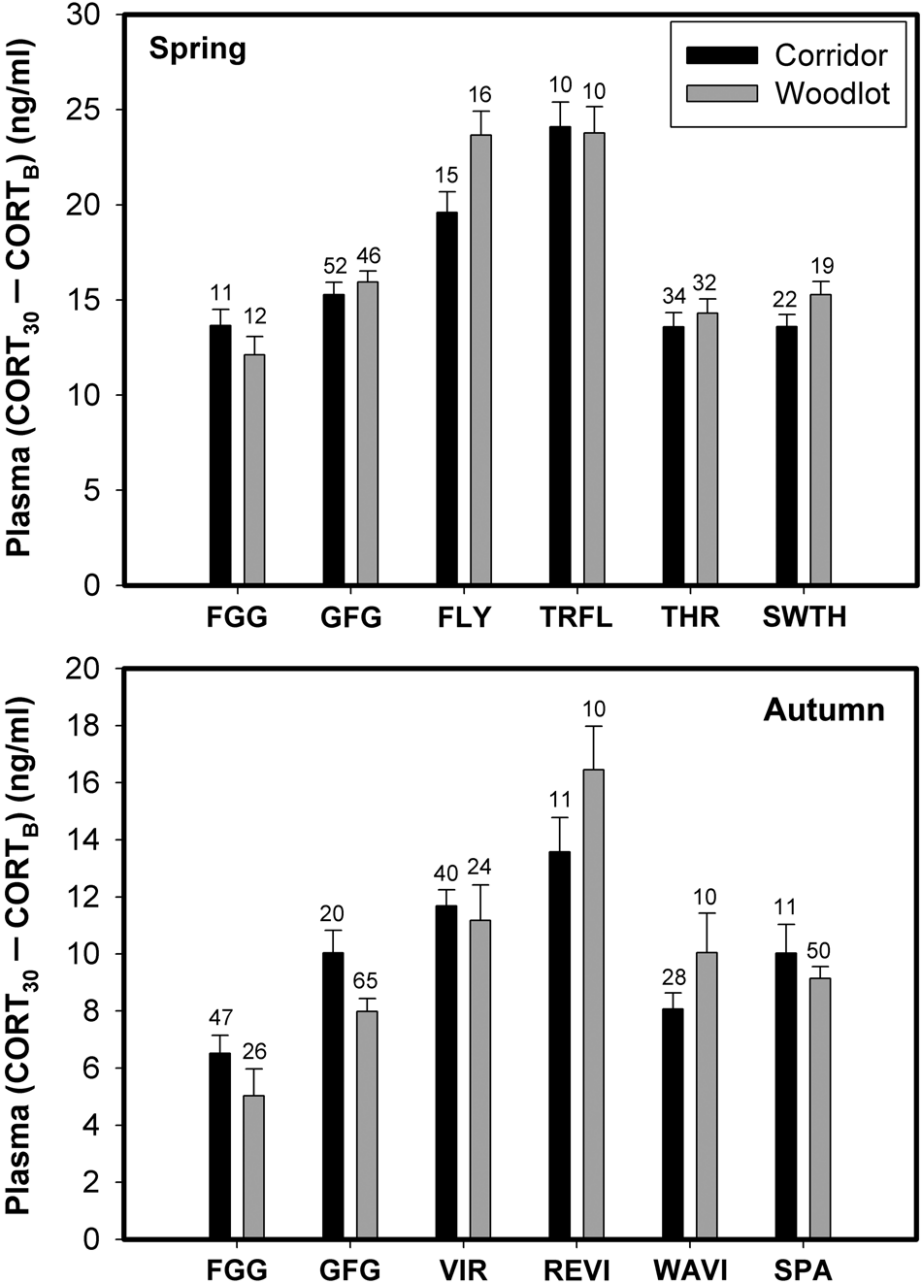
Mean (±SEM) magnitude of the stress response (CORT_30_ − CORT_B_, in nanograms per millilitre) for spring and autumn individual species, taxa and foraging guilds. Abbreviations are as in Tables 1 and 2.

No individual species, taxon or foraging guild (see Table 1 for list of species captured) showed significant differences in CORT_B_ between habitat types for either spring or autumn migration (Fig. 2). All individual species, taxa and foraging guilds in both habitats showed significantly elevated plasma CORT after 30 min of restraint relative to baseline levels, indicating that all birds were capable of mounting a stress response during spring and autumn migration (Figs 2 and 3, Supplementary Table 1). The CORT_30_ did not vary significantly between birds from the two habitats for any group (Supplementary Table 1). Moreover, we did not detect any significant between-habitat differences in the magnitude of the stress response for any individual species, taxon or foraging guild from either spring or autumn migration (Fig. 3). During autumn migration, both adults and juveniles showed significant stress responses. However, no significant between-age differences occurred for CORT_B_, CORT_30_ or the magnitude of stress response for any individual species, taxon or foraging guild.

We found no significant species effects for between-habitat comparisons for either foraging guilds or taxa in spring. In autumn, a significant species effect occurred for CORT_B_ only for the sparrow group (*F*_5,50_ = 3.32, *P* = 0.01) and for the magnitude of the stress response only for the foliage-gleaning guild (*F*_5,60_ = 3.21, *P* = 0.02). A significant species effect indicates a strong influence of particular species on plasma CORT values for the taxon or foraging group as a whole. After between-habitat comparisons were adjusted statistically for these significant species effects, between-habitat comparisons of CORT_B_, CORT_30_ and the magnitude of the stress response remained non-significant for these bird groups. The interaction between species and habitat type for comparisons of plasma CORT was not significant for any foraging guild or taxon in either season.

### Effects of covariates on CORT levels

Several covariates influenced plasma CORT levels, for CORT_B_, CORT_30_ or for the magnitude of stress response. The CORT_B_ was significantly higher in the autumn of 2011 than in the autumn of 2010 for all migrant groups tested, except for red-eyed vireos (*Vireo olivaceus*), which showed no significant effect of sampling year on CORT_B_ (Table 2), but year was not a significant effector of CORT levels in other comparisons. Fat scores showed a significant positive correlation with CORT_B_ and significant negative correlations with CORT_30_ and the magnitude of the stress response for the ground-foraging guild, warbling vireos and sparrows during autumn migration (Table 2). Body size (described by the principal component analysis) did not exert a significant influence on plasma CORT for most bird groups, with the exception of the ground-foraging guild in spring and the foliage-gleaning guild and vireos in autumn, where smaller birds showed higher CORT_B_ and a lower CORT_30_ and magnitude of the stress response (Table 2). Autumn migrants did not show any significant difference in CORT_B_, CORT_30_ or the magnitude of the stress response between juveniles and adults. We found no significant effects of capture date or time on CORT_B_, CORT_30_ or the magnitude of the stress response for any individual species, taxon or foraging guild during either spring or autumn migration, except for a positive effect of capture date on the magnitude of the stress response in autumn migrant vireos, where vireos captured later in the season showed a greater stress response (Table 2). The CORT_B_ was significantly higher in spring than in autumn only for the ground-foraging guild. The CORT_30_ and the magnitude of the stress response were significantly higher in spring than in autumn for both foliage-gleaning and ground-foraging guilds.

**Table 2:** Variables retained in backward stepwise multiple regression models for plasma corticosterone at the *P* < 0.10 level for individual species, taxa and foraging guilds

Guilds/species	Spring			Autumn					
	CORT_B_	CORT_30_	CORT_30_ − CORT_B_	CORT_B_		CORT_30_		CORT_30_ − CORT_B_	
FGG	None	None	None	PC −	Y +	PC +		PC +	
GFG	PC −	PC +	PC +	Y +	F +	F −		F −	
THR	None	None	None						
SWTH	None	None	None						
FLY	None	None	None						
TRFL	None	None	None						
VIR				PC −	D +	PC +	D +	PC +	D +
REVI				None		None		None	
WAVI				Y +	F +	F −		F −	
SPA				Y +	F +	F −		F −	

Positive or negative symbols indicate direction of effect. ‘None’ means that no covariate showed significant effects on plasma corticosterone levels. Abbreviations are as in Table 1, plus the following: D, capture date; F, fat score; PC, principal component for body size; REVI, red-eyed vireo, *Vireo olivaceus*; SWTH, Swainson's thrush, *Catharus ustulatus*; T, capture time; TRFL, complex of *Empidonax alnorum* and *Empidonax traillii*; WAVI, warbling vireo, *Vireo gilvus*; Y, year.

### Relationships among CORT_B_, stress response and plasma metabolites

**Figure 4:**
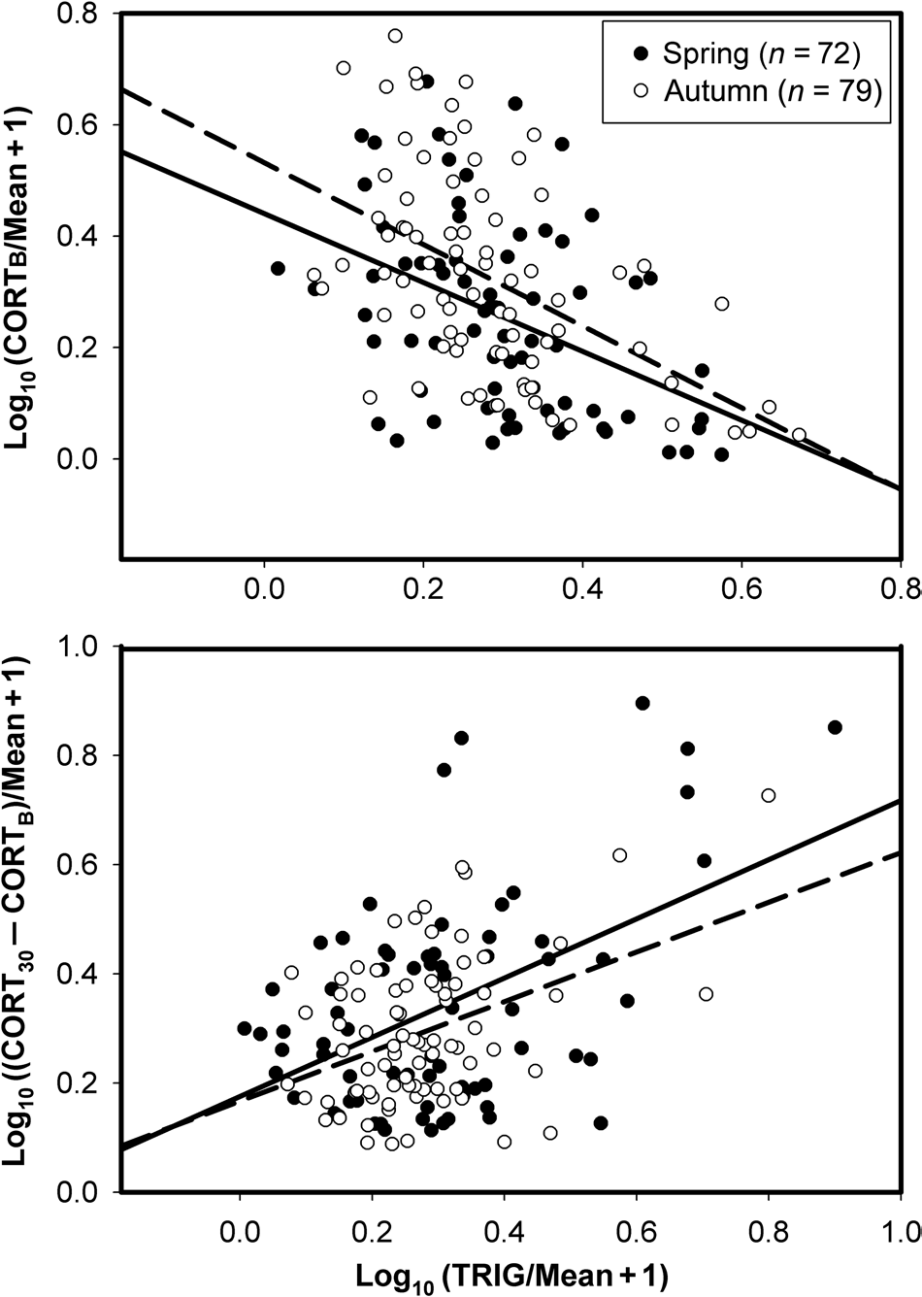
Relationship of baseline corticosterone (CORT_B_) and the magnitude of the stress response (CORT_30_ − CORT_B_) with plasma triglycerides (TRIG). The dashed line represents autumn migrants and the continuous line spring migrants. The CORT_B_ showed a significant negative correlation with TRIG in both seasons [spring, CORT_B_ = 0.44 − 0.62(TRIG); and autumn, CORT_B_ = 0.53 − 0.73(TRIG)]. The magnitude of the stress response showed a significant positive correlation with TRIG in both seasons [spring, CORT_30_ − CORT_B_ = 0.18 + 0.54(TRIG); and autumn, CORT_30_ − CORT_B_ = 0.17 + 0.46(TRIG)].

**Figure 5:**
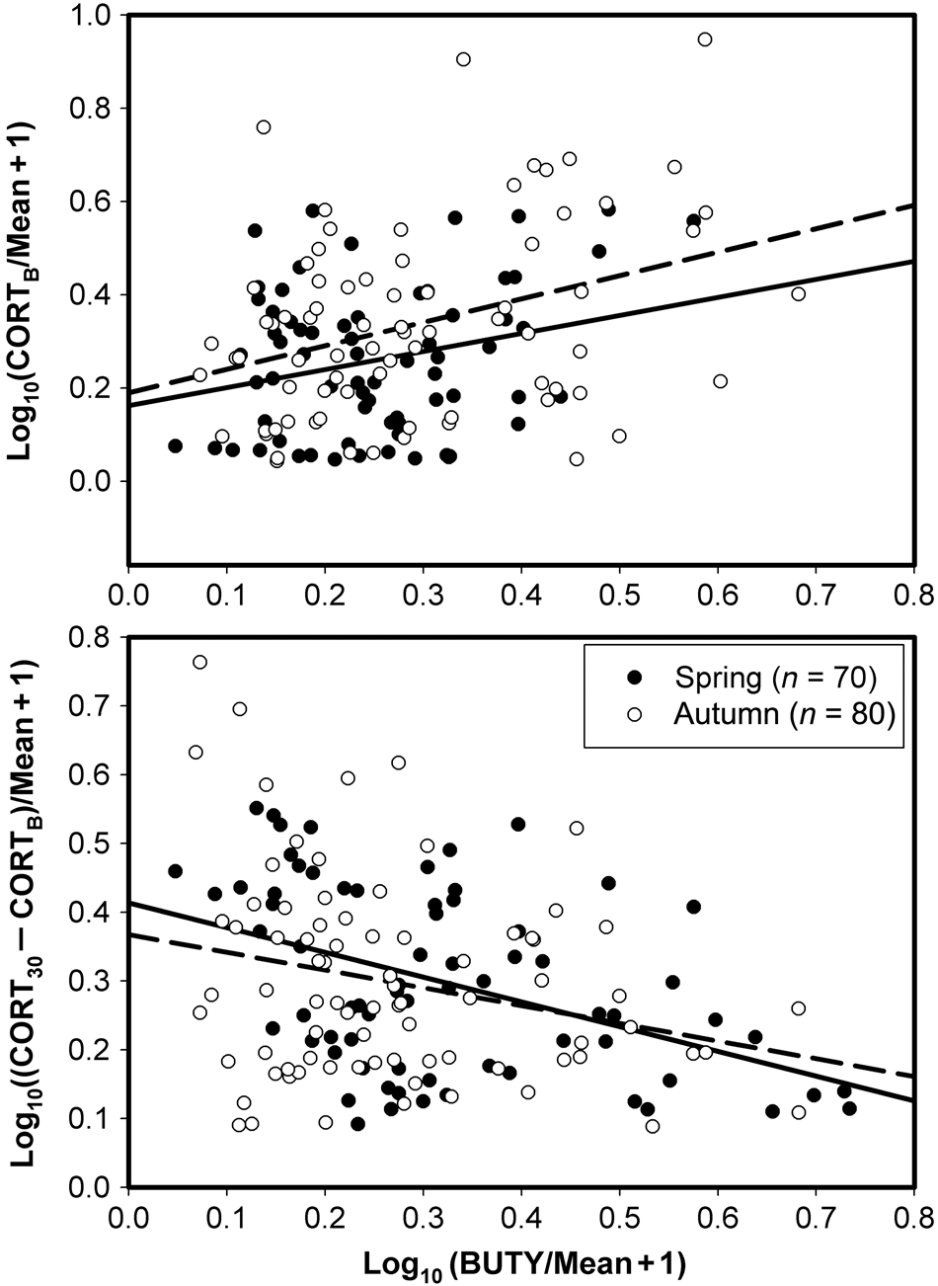
Relationship of baseline corticosterone (CORT_B_) and the magnitude of the stress response (CORT_30_ − CORT_B_) with plasma β-hydroxybutyrate (BUTY). The dashed line represents autumn migrants and the continuous line spring migrants. The CORT_B_ showed a significant positive correlation with BUTY in both seasons [spring, CORT_B_ = 0.16 + 0.39(BUTY); and autumn, CORT_B_ = 0.19 + 0.50(BUTY)]. The magnitude of the stress response showed a significant negative correlation with BUTY in both seasons [spring, CORT_30_ − CORT_B_ = 0.41 − 0.36(BUTY); and autumn, CORT_30_ − CORT_B_ = 0.37 − 0.26(BUTY)].

**Figure 6:**
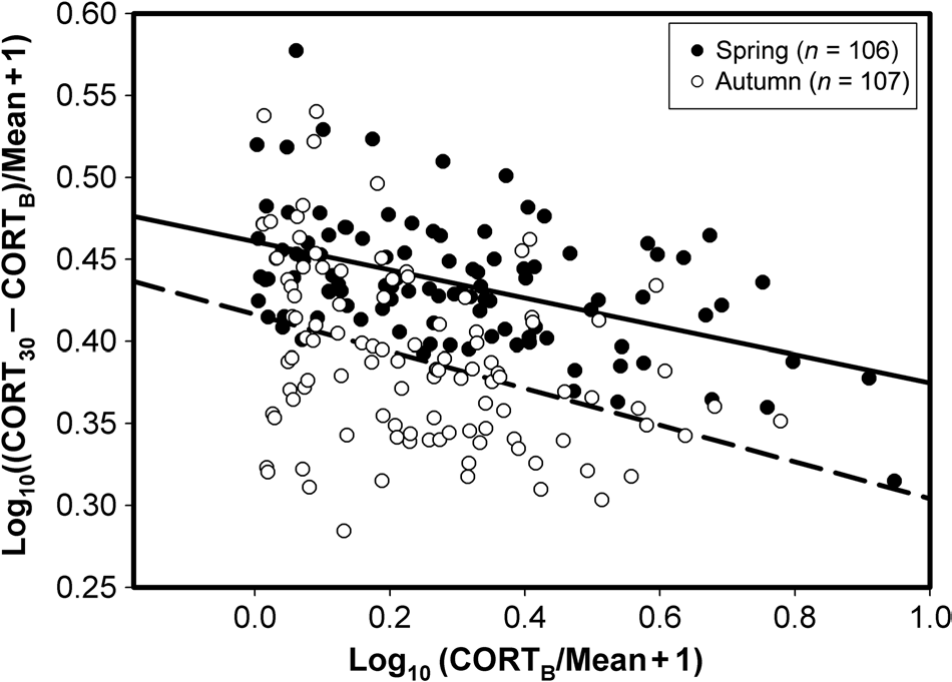
Relationship between baseline corticosterone (CORT_B_) and the magnitude of the stress response (CORT_30_ − CORT_B_). The dashed line represents autumn migrants and the continuous line spring migrants. Significant negative correlations occurred between these variables in both seasons [spring, CORT_30_ − CORT_B_ = 0.46 − 0.09(CORT_B_); and autumn, CORT_30_ − CORT_B_ = 0.42 − 0.11(CORT_B_)].

Normalized CORT_B_ showed a significant negative correlation with normalized plasma TRIG (spring, *F*_1,69_ = 16.05, *r* = 0.43; and autumn, *F*_1,76_ = 26.38, *r* = 0.50; *P* < 0.001 in both cases), and the normalized magnitude of the stress response showed a significant positive correlation with normalized plasma TRIG (spring, *F*_1,69_ = 21.83, *r* = 0.49; and autumn, *F*_1,76_ = 16.00, *r* = 0.41; *P* < 0.001 in both cases; Fig. 4). Normalized CORT_B_ showed a significant positive relationship to normalized plasma BUTY (spring, *F*_1,67_ = 4.93, *r* = 0.26, *P* = 0.03; and autumn, *F*_1,77_ = 10.49, *r* = 0.35, *P* = 0.002), and the normalized magnitude of the stress response showed a significant negative relationship to normalized plasma BUTY (spring, *F*_1,67_ = 16.13, *r* = 0.44, *P* < 0.001; and autumn, *F*_1,77_ = 16.05, *r* = 0.25, *P* = 0.02; Fig. 5). Normalized CORT_B_ and the magnitude of the stress response showed significant negative correlations in both spring (*F*_1,103_ = 31.23, *P* < 0.001, *r* = 0.48) and autumn migrations (*F*_1,104_ = 16.74, *P* < 0.001, *r* = 0.37; Fig. 6).

## Discussion

Anthropogenic woodlots might be expected to provide less favourable stopover habitats for landbird migrants than natural riparian corridor woodlands because of their smaller size, increased isolation, increased edge and lower vegetative diversity (Castonguay, 1982; Swanson *et al.*, 2003; Gentry *et al.*, 2006). As a consequence of these differences, migrants stopping over in woodlots could be exposed to different levels of food availability, competition intensity, predation pressure and thermal microclimates than migrants stopping over in corridors, and these factors might lead to differences in stress physiology for birds in woodlots and corridors (Clinchy *et al.*, 2004; Kitaysky *et al.*, 2007; Olson and Grubb, 2007; Olson *et al.*, 2010; Braasch *et al.*, 2014). In the present study, however, we found no significant differences for any CORT variables (CORT_B_, CORT_30_ or the magnitude of the stress response) between birds captured in corridors and woodlots, suggesting that the stress physiology of migrant birds in both corridors and woodlots is generally similar. In addition, all individual species, taxa and foraging guilds from both spring and autumn migration showed a significant stress response after a 30 min restraint stress, which suggests that birds were in sufficient body condition in both woodland habitats to mount a robust stress response (i.e. a significant increase in plasma CORT with restraint stress). These data suggest that the increased reliance of migrating birds on anthropogenic woodlands with the loss and degradation of natural riparian corridor habitats in the Northern Prairie region, where woodland habitat is scarce, does not negatively impact their stress physiology. This result is consistent with the finding of similar refuelling rates and body condition between birds in the two habitat types at these same study sites (Liu and Swanson, 2014). Collectively, these data suggest similar habitat quality in the two habitat types and support the idea that anthropogenic woodlot habitats can, at least partly, substitute for lost and degraded riparian corridor woodlands as stopover habitat for migrant birds in the Northern Prairie region of North America.

Among the covariates influencing plasma CORT levels in our study, sampling year significantly affected CORT_B_ for several bird groups in autumn, and CORT_B_ levels were higher in 2011 than in 2010 for all of these groups. This result suggests that ecological or environmental conditions (e.g. food availability, predation pressure, intensity of competition, weather) may vary among years at our study sites in a manner that influences stress physiology of migrants. Defining which, if any, of these factors might be associated with the elevated CORT_B_ levels in 2011 relative to 2010 will require further study, but two factors potentially related to this pattern bear mentioning. First, a large and prolonged flood impacted the Missouri River throughout the summer and early autumn of 2011 (Munes, 2014). In addition, autumn 2011 was considerably drier than autumn 2010 near Vermillion, Clay County, southeastern South Dakota, with precipitation in September and October of 2011 only 39% of that during the same months in 2010 (US Climate data, http://www.usclimatedata.com/). Liu and Swanson (2014) detected lower plasma TRIG levels in autumn 2011 than in autumn 2010 for several migrant bird groups at these same study sites, suggesting that conditions in autumn 2011 resulted in lower refuelling rates than in autumn 2010, which is consistent with relatively lower quality stopover habitat resulting in elevated CORT_B_ levels in autumn 2011.

Body size may also be related to plasma CORT_B_ levels in birds, with smaller birds exhibiting higher baseline levels than larger birds (Lehnen and Krementz, 2005). In the present study, the principal component describing body size had a significant effect on plasma CORT levels for only a few bird groups. The general absence of an effect of body size on plasma CORT in migrating birds is consistent with the findings of Thomas (2008), who documented that the body size of migrating shorebirds did not influence the overall CORT response. However, for those bird groups in our study where body size was significantly related to plasma CORT levels (ground-foraging guild in spring and foliage-gleaning guild and vireos in autumn), body size was negatively related to CORT_B_ and positively related to the magnitude of the stress response. This result is consistent with the relationship documented by Lehnen and Krementz (2005) and suggests that larger birds are in better condition than and/or outcompete smaller individuals for resources in these bird groups. We have no obvious explanation for why such a relationship occurs for only some bird groups and not for others, but perhaps differential use of or access to resources is involved.

Hatch-year migrants are undertaking their first migration in autumn, so they are less experienced foragers than adults, which could result in lower refuelling rates, although age-related differences in body condition and refuelling rates are seldom detected at stopover sites (e.g. Seewagen *et al.*, 2013; Liu and Swanson, 2014). Moreover, juvenile migrants are usually socially subordinate to adults, which may restrict the former's access to limited resources due to competitive interactions with experienced adults (Yong *et al.*, 1998; Heise and Moore, 2003; Moore *et al.*, 2003; Faaborg *et al.*, 2010). As a consequence, the stress response may differ between age classes, with higher CORT_B_ and a lower stress response in juveniles than in adults. In the present study, we did not detect significant differences in CORT_B_, CORT_30_ or the magnitude of the stress response between adults and juveniles. This is similar to the results of Thomas (2008), who found little difference in plasma CORT levels between adult and juvenile least (*Calidris minutilla*) and semipalmated sandpipers (*Calidris pusilla*). This suggests that ecological and environmental conditions at our stopover sites provided effective stopover habitat for both adults and juvenile birds such that age-related differences in stress physiology were not evident.

We obtained sufficient samples sizes for between-season comparisons only for foliage-gleaning and ground-foraging guilds and found that both groups had higher CORT_B_ and a greater magnitude of the stress response in the spring compared with autumn migration. Spring migration is potentially more urgent than autumn, because early arrival on the breeding grounds generally shows a positive correlation with fitness (e.g. Smith and Moore, 2005; Cooper *et al.*, 2011; Gargallo *et al.*, 2011; Gienapp and Bregnballe, 2012). Spring migrants typically migrate at a faster pace than autumn migrants (Pearson and Lack, 1992; Ellegren, 1993; Fransson, 1995; Kemp *et al.*, 2010), which may reflect differences in their physiological capacities (Swanson, 1995; Swanson and Dean, 1999). These differences might result in higher CORT_B_ in spring than in autumn migrants and promote greater foraging intensity and better refuelling performance than for birds during the more leisurely autumn migration. Such an explanation is consistent with our previous study suggesting better refuelling performance in spring migrants than in autumn migrants at these same study sites (Liu and Swanson, 2014).

The migration modulation hypothesis proposes that migrants maintain elevated CORT_B_ levels during migration and show a suppressed stress response when faced with additional acute stressors (Holberton *et al.*, 1996), thereby facilitating fattening while sparing muscle protein. Consistent with this hypothesis, elevated CORT_B_ levels, a suppressed stress response, or both, occur in a number of wild migrant or photostimulated, migratory-ready captive birds (Holberton *et al.*, 1996, 2007; Piersma *et al.*, 2000; Landys-Ciannelli *et al.*, 2002; Long and Holberton, 2004). However, numerous studies also document significant stress responses in wild migrant or photostimulated captive birds (Romero *et al.*, 1997; Piersma *et al.*, 2000; Landys-Ciannelli *et al.*, 2002; Falsone *et al.*, 2009; Nilsson and Sandell, 2009), suggesting that full suppression of the stress response does not occur in most migrants or that the degree of suppression changes with the stage of migratory stopover. However, the magnitude of the stress response in these birds may sometimes be influenced by body condition (Mizrahi *et al.*, 2001; Jenni-Eiermann *et al.*, 2009; Raja-aho *et al.*, 2010), so energetic condition could influence the modulation of the stress response during migration. A corollary prediction of the migration modulation hypothesis is that CORT_B_ and the magnitude of the stress response should be inversely related, even if the stress response is not fully suppressed. The suppressed stress response when facing additional acute stressors might result from negative feedback of elevated CORT_B_ during migration (Raja-aho *et al.*, 2010). In the present study, we found a significant negative relationship between CORT_B_ and the magnitude of the stress response, despite robust stress responses for all bird groups studied, which is consistent with this corollary prediction of the migration modulation hypothesis.

High plasma CORT concentrations may promote elevated levels of gluconeogenesis and lipogenesis, leading to fat deposition, while also stimulating protein catabolism (Holmes and Phillips, 1976; Berdanier, 1989; but see Eikenaar *et al.*, 2013, 2014). Plasma levels of TRIG and BUTY are considered the best metabolites for plasma metabolite profiling in birds (Guglielmo *et al.*, 2002, 2005; Smith and McWilliams, 2010; Seewagen *et al.*, 2011) and they are now routinely used to measure fattening rates of birds during migration because plasma TRIG increases during fat deposition and plasma BUTY increases during fat catabolism (Schaub and Jenni, 2001; Seaman *et al.*, 2005; Cerasale and Guglielmo, 2006, 2010). We found a significant negative correlation of CORT_B_ and a significant positive correlation of the magnitude of the stress response with plasma TRIG for birds in the present study. Plasma BUTY showed significant opposite patterns of correlations with CORT_B_ and the magnitude of the stress response in comparison to plasma TRIG. These data support our hypotheses that high-quality habitat promotes effective fattening and reduced CORT_B_, while allowing a robust stress response.

Furthermore, CORT measures can potentially provide additional information regarding the relationships among the stress response, fattening, body condition and stopover biology in migrants. Plasma CORT shows a U-shaped relationship with fat stores in shorebirds (Piersma *et al.*, 2000), and Eikenaar *et al.* (2013) found a positive correlation between plasma CORT and fat stores before departure for a passerine bird, the northern wheatear (*Oenanthe oenanthe*). If landbird migrants also show a U-shaped relationship between plasma CORT and fat stores during stopover, but a linear relationship with plasma metabolites (present study), analyses including CORT, plasma metabolites and fat stores might allow teasing apart of the effects of different stopover durations prior to capture on plasma metabolite comparisons. The U-shaped relationship between plasma CORT_B_ with stopover duration in shorebirds (Piersma *et al.*, 2000) suggests that migrants have high CORT_B_ levels upon arrival at a stopover site and then show a decline as their body condition improves, only to increase CORT again before departure, when fat stores are high (e.g. Eikenaar *et al.*, 2013). Such a pattern might be driven by differential sensitivity of receptors to CORT at different CORT levels or by regulation of the hypothalamo–pituitary–adrenal axis at the brain level (e.g. de Kloet *et al.*, 1999; Matthews, 2002; Sapolsky, 2002). As a consequence, migrating birds may show considerable variation in the relationship between CORT_B_ and fat stores. Indeed, negative relationships (Long and Holberton, 2004; Raja-aho *et al.*, 2010), positive relationships (Romero *et al.*, 1997; Holberton, 1999, Landys-Ciannelli *et al.*, 2002; Landys *et al.*, 2004; Eikenaar *et al.*, 2013) and no relationship (Holberton *et al.*, 1996; Mizrahi *et al.*, 2001) between CORT_B_ and fat stores or energetic condition have been documented among migratory birds. We found no significant relationships between fat scores and CORT_B_ for any individual species, taxon or foraging guild during spring migration. During autumn migration, fat score was a significant covariate in multiple regression models for several bird groups and showed a consistent positive relationship with CORT_B_ and negative relationship with the magnitude of the stress response in these groups. One possible explanation for these results is that fat birds within these groups were ready to migrate, and elevated CORT_B_ levels and a smaller stress response prior to departure served to prepare them for upcoming behavioural and metabolic transitions during migratory flights, similar to the suggestion of Piersma *et al.* (2000) for shorebirds and Eikenaar *et al.* (2013) for northern wheatears. Tracking these variables concurrently during stopover might allow more precise estimation of the stage of migratory stopover from a single blood sample than has been possible to date. However, validation of the potential for this method will require much additional research and larger sample sizes for individual species than those in the present study.

## Supplementary material

Supplementary material is available at *Conservation Physiology* online.

## Funding

This study was funded by grants from the US Fish and Wildlife Service (Award Number F10AC00572), University of South Dakota and South Dakota Ornithologists' Union (Nathaniel R. Whitney Jr. Memorial Research Grant).

## Supplementary Material

Supplementary Data
