# Supplementary material for: Stress physiology of migrant birds during stopover in natural and anthropogenic woodland habitats of the Northern Prairie region
Source: Conserv Physiol. 2014 Oct 11;2(1):cou046. doi: 10.1093/conphys/cou046 (PMC4806743; doi:10.1093/conphys/cou046)
Supplement: Supplementary Data [file supp_2_1_cou046__index.html]

Supplementary Data 

# Stress physiology of migrant birds during stopover in natural and anthropogenic woodland habitats of the Northern Prairie region

## Supplementary Data

Supplementary Data

**Files in this Data Supplement:**

- Supplementary Table 1 - docx file
