# Supplementary material for: Stress physiology of migrant birds during stopover in natural and anthropogenic woodland habitats of the Northern Prairie region
Source: Conserv Physiol. 2014 Oct 11;2(1):cou046. doi: 10.1093/conphys/cou046 (PMC4806743; doi:10.1093/conphys/cou046)
Supplement: Supplementary Data [file supp_cou046_cou046supp_table1.docx]

si Table 1: Mean (±SE) corticosterone concentration (ng/ml) and sample sizes (in parentheses) for individual species, taxa, and foraging guilds by stopover habitat and season. Abbreviations: CO = Corridors, WO = Woodlots; CORT_B_ = baseline corticosterone, CORT_30_ = corticosterone after a 30-min restraint stress; FGG = foliage-gleaning guild, GFG = ground-foraging guild; VIR = vireos, THR = thrushes, SPA = sparrows, FLY = flycatchers; TRFL = complex of *Empidonax alnorum* and *Empidonax traillii*; REVI = Red-eyed Vireo, *Vireo olivaceus*; WAVI = Warbling Vireo, *Vireo gilvus*; SWTH = Swainson’s Thrush, *Catharus ustulatus*.

| Seasons | Guild/taxon/species | CORT_B_ | |  | CORT_30_ | |
| --- | --- | --- | --- | --- | --- | --- |
|  |  | CO | WO |  | CO | WO |
| Spring | FGG | 5.77±1.43 (11) | 4.87±1.41 (12) |  | 19.42±1.12 (11) | 16.99±1.47 (12) |
|  | GFG | 4.04±0.80 (52) | 4.98±0.30 (46) |  | 19.32±1.16 (52) | 20.92±1.46 (46) |
|  | FLY | 4.64±1.04 (15) | 5.19±1.52 (16) |  | 24.24±2.20 (15) | 28.84±2.28 (16) |
|  | TRFL | 4.41±1.07 (10) | 4.01±1.22 (10) |  | 28.50±2.84 (10) | 27.78±2.95 (10) |
|  | THR | 2.36±0.45 (34) | 3.36±0.52 (32) |  | 15.94±1.80 (34) | 17.66±1.73 (32) |
|  | SWTH | 2.97±0.42 (22) | 3.41±0.54 (19) |  | 16.57±1.47 (22) | 18.68±1.56 (19) |
| Fall | FGG | 4.65±0.72 (47) | 4.91±1.45 (26) |  | 11.17±1.17(47) | 9.94±1.37 (26) |
|  | GFG | 2.78±1.03 (20) | 2.50±0.37 (65) |  | 12.82±1.33 (20) | 10.49±0.98 (65) |
|  | VIR | 3.12±0.43 (40) | 2.16±0.86 (24) |  | 14.81±1.26 (40) | 13.34±2.85 (24) |
|  | REVI | 2.78±1.34 (11) | 2.62±1.44 (10) |  | 16.36±2.24 (11) | 19.07±3.14 (10) |
|  | WAVI | 5.27±0.86 (28) | 4.92±1.26 (10) |  | 13.34±0.84 (28) | 14.97±2.89 (10) |
|  | SPA | 1.36±0.79 (11) | 1.09±0.21 (50) |  | 11.39±2.22 (11) | 10.24±1.02 (50) |
